# miR-6745-TIMP1 axis inhibits cell growth and metastasis in gastric cancer

**DOI:** 10.18632/aging.203688

**Published:** 2021-11-14

**Authors:** Hui Liu, Yuan Xiang, Qi-Bei Zong, Xiao-Yu Zhang, Zhi-Wen Wang, Shi-Qiang Fang, Tong-Cun Zhang, Xing-Hua Liao

**Affiliations:** 1 Institute of Biology and Medicine, College of Life and Health Sciences, Wuhan University of Science and Technology, Hubei 430081, P.R. China; 2 Department of Medical Laboratory, Central Hospital of Wuhan, Tongji Medical College, Huazhong University of Science and Technology, Hubei 430014, P.R. China; 3 Yueyang Key Laboratory of Chronic Noncommunicable Diseases, Yueyang Vocational and Technical College, Hunan 414000, P.R. China

**Keywords:** TIMP1, miR-6745, oncogenesis, Wnt/β-catenin pathway, gastric cancer

## Abstract

Tissue inhibitor matrix metalloproteinase 1 (TIMP1) has been reported to act as a tumor oncogene in colon cancer. However, little is known about the biological role of TIMP1 in gastric cancer. In this study, we found that the expression of TIMP1 in GC tissues was upregulated compared with the normal gastric tissues. TIMP1 was confirmed as a direct target of miR-6745 and silencing TIMP1 mimicked the effects of miR-6745 in GC cells. Further mechanism studies have shown that miR-6745 inhibits the Wnt/β-catenin pathway by targeting TIMP1, thereby inhibiting cell proliferation, migration and invasion. In addition, through the analysis of GC tissues, a negative correlation between miR-6745 and TIMP1 was found in 42 GC tissues. Our findings indicate that the miR-6745-TIMP1 axis regulates Wnt/βcatenin signaling and participates in GC tumorigenesis and provide a potential therapeutic target for preventing GC progression.

## INTRODUCTION

Gastric cancer (GC) is a frequently occurring malignant cancer [1, 2]. According to statistics from the World Health Organization, there are approximately 1 million new cases of GC patients every year, and GC has become the third leading cause of cancer deaths worldwide [2, 3]. Although an increasing number of GC diagnosis and treatment strategies have been developed in recent years, the prognosis of patients is still very poor [4, 5]. Thus, it is important to elucidate the molecular mechanism of the occurrence and development of GC.

MicroRNA is an endogenous small non-coding RNA that can regulate protein expression levels [6, 7]. It has been reported that miRNAs play important biological functions in various types of human cancers, including oncogenes or tumor suppressor genes [8, 9]. Exploring the molecular mechanisms that miRNA regulates the occurrence and development of cancer will help the treatment and diagnosis of tumors.

Tissue inhibitor matrix metalloproteinase 1 (TIMP1) is one of the tissue inhibitor members of the metalloproteinase family [10]. TIMP1 regulates the balance of matrix remodeling during the degradation of extracellular matrix by inhibiting the proteolytic activity of matrix metalloproteinases (MMPs) [10, 11]. Studies have shown that TIMPs also perform important biological functions in cell proliferation and metastasis [10, 12–14]. In clinical studies, the high expression of TIMP-1 in the serum of patients with various tumors is often associated with poor prognosis [15–17]. However, the molecular mechanism of TIMP1 in GC remain to be elucidated.

Our research is to explore the biological function of miR-6745 in the development of GC. miR-6745 regulates the expression of TIMP1 to inhibit cell growth and reduce the ability of metastasis *in vitro* and *in vivo.* In addition, we proved the role of miR-6745/TIMP1/Wnt/β-catenin signaling in the development of GC.

## RESULTS

### TIMP1 is upregulated in human gastric cancer

**Figure 1:**
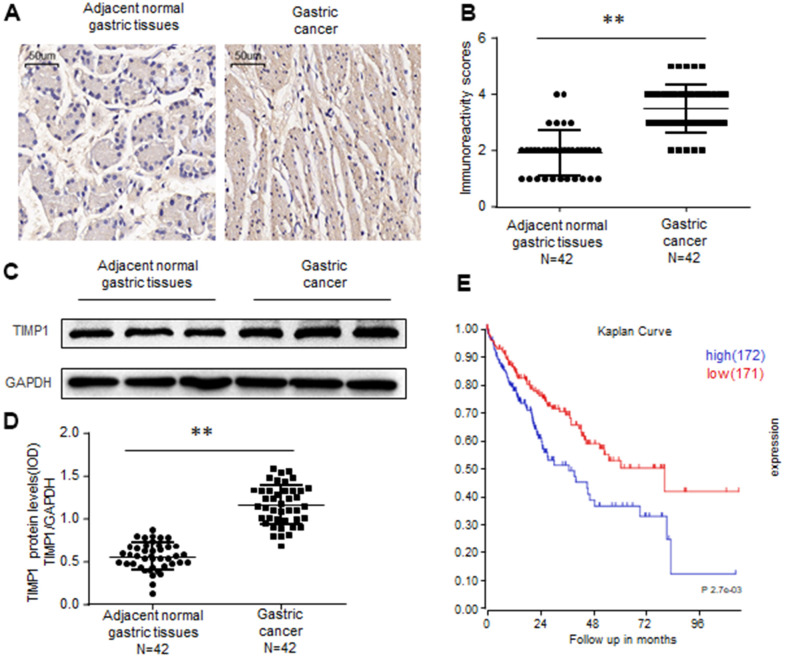
**TIMP1 is upregulated in human gastric cancer.** (**A**, **B**) Immunohistochemistry of TIMP1 expression in 42 gastric carcinoma tissues and 42 adjacent normal gastric tissues. Representative immunohistochemistry images (**A**) and semi-quantitative evaluation (**B**) of TIMP1protein expression. (**C**, **D**) Analysis of TIMP1 expression in 42 gastric carcinoma tissues and 42 adjacent normal gastric tissues. Representative western blotting images of TIMP1 protein levels in three normal gastric tissues and three gastric carcinoma tissues (**C**). TIMP1 and GAPDH protein levels were determined via densitometry using ImageJ and are represented as IOD (**D**). (**E**) Kaplan-Meier survival analyses of GC patients with high or low TIMP1 expression based on KM plotter database and GSE15459 dataset Data represent the means ± SEM. **P < 0.01. ns, not significant.

To investigate whether TIMP1 is upregulated in Gastric Cancer (GC), we first examined TIMP1 expression in 42 cases of GC and 42 adjacent normal gastric tissues. Immunochemistry revealed that, compared with control colon tissues TIMP1 was upregulated in gastric cancer tissues (Figure 1A, 1B). By testing the frozen gastric tissues, we also found that TIMP1 increased in protein levels (Figure 1C, 1D). Kaplan-Meier analysis showed that high levels of TIMP1 expression are associated with poor overall survival rates of GC patients. In summary, these findings indicate that TIMP1 expression is upregulated in GC tissues.

### TIMP1 promotes GC proliferation, migration and invasion

To explore the biological function of TIMP1 in the development of GC, GC cells were transduced with siRNAs targeting TIMP1 or TIMP1 overexpression plasmid. Our results showed that, compared with control cells, the proliferation ability was significantly improved in TIMP1 overexpression cells (Figure 2A–2C), and inhibited in TIMP1 knockdown cells (Supplementary Figure 1A–1C). Wound healing assay and Transwell showed that, TIMP1 promotes GC migration and invasion (Figure 2D, 2E and Supplementary Figure 1D, 1E). Increased expression of Ki67 indicated that TIMP1 significantly promoted GC cell proliferation (Figure 2F). Subsequently, inhibiting the expression of TIMP1 can also cause a decrease in the expression of Ki67 (Supplementary Figure 2F).

**Figure 2a:**
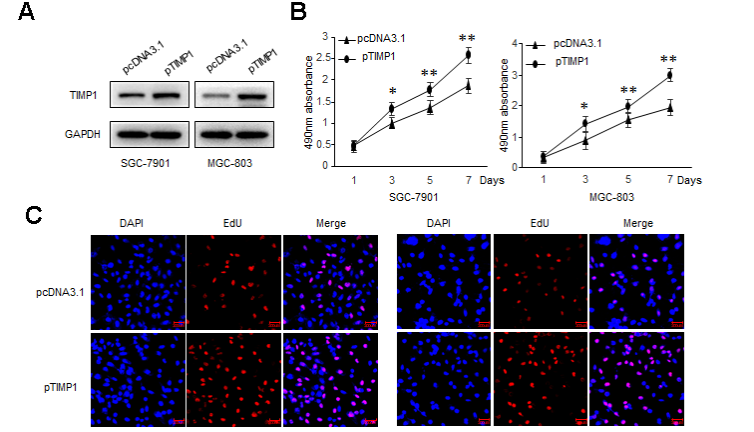
**TIMP1 promotes GC proliferation, migration and invasion.** SGC-7901 and MGC-803 cells were transduced with TIMP1 expression plasmid (pTIMP1) or pcDNA3.1 as indicated. (**A**) Levels of TIMP1 were detected by western blot. (**B**) Cell proliferation was determined at the indicated time points by MTS assay. (**C**) Effect of TIMP1 on cell proliferative abilities was examined by EdU incorporation assay.

**Figure 2b:**
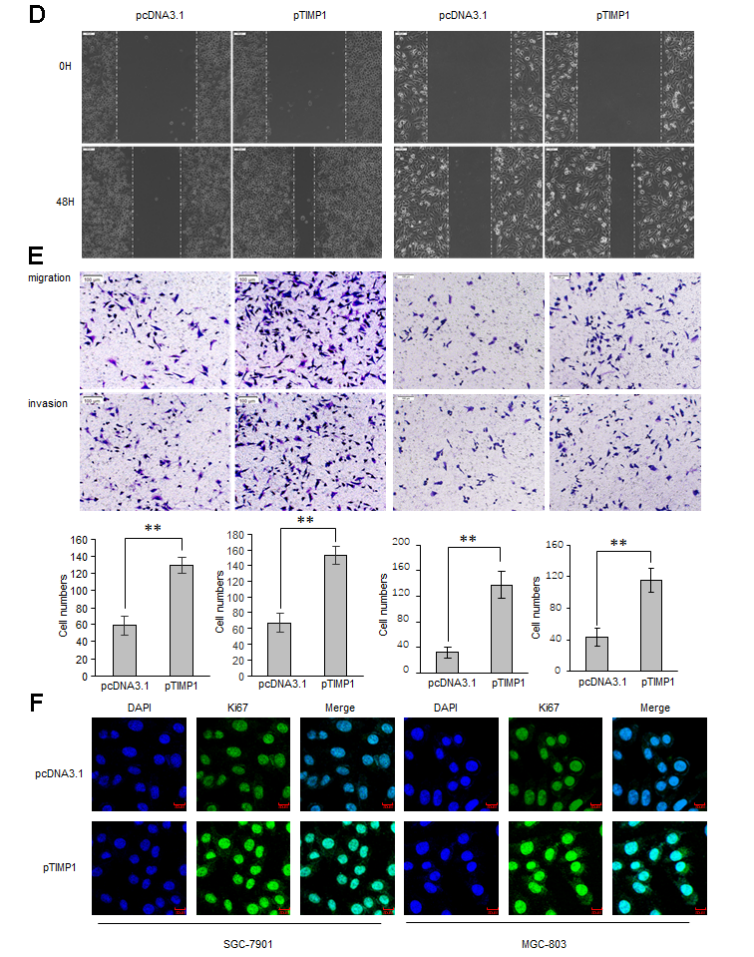
**TIMP1 promotes GC proliferation, migration and invasion.** SGC-7901 and MGC-803 cells were transduced with TIMP1 expression plasmid (pTIMP1) or pcDNA3.1 as indicated. (**D**, **E**) Cell metastasis was determined by Scratch wound assays (**D**) or Transwell migration and Matrigel invasion assays (**E**). (**F**) The expression levels of the cell proliferation marker Ki67 were detected by immunofluorescence. Data represent the means ± SEM. **P < 0.01.

### miR-6745 directly binds to the TIMP1 3'-UTR

**Figure 3:**
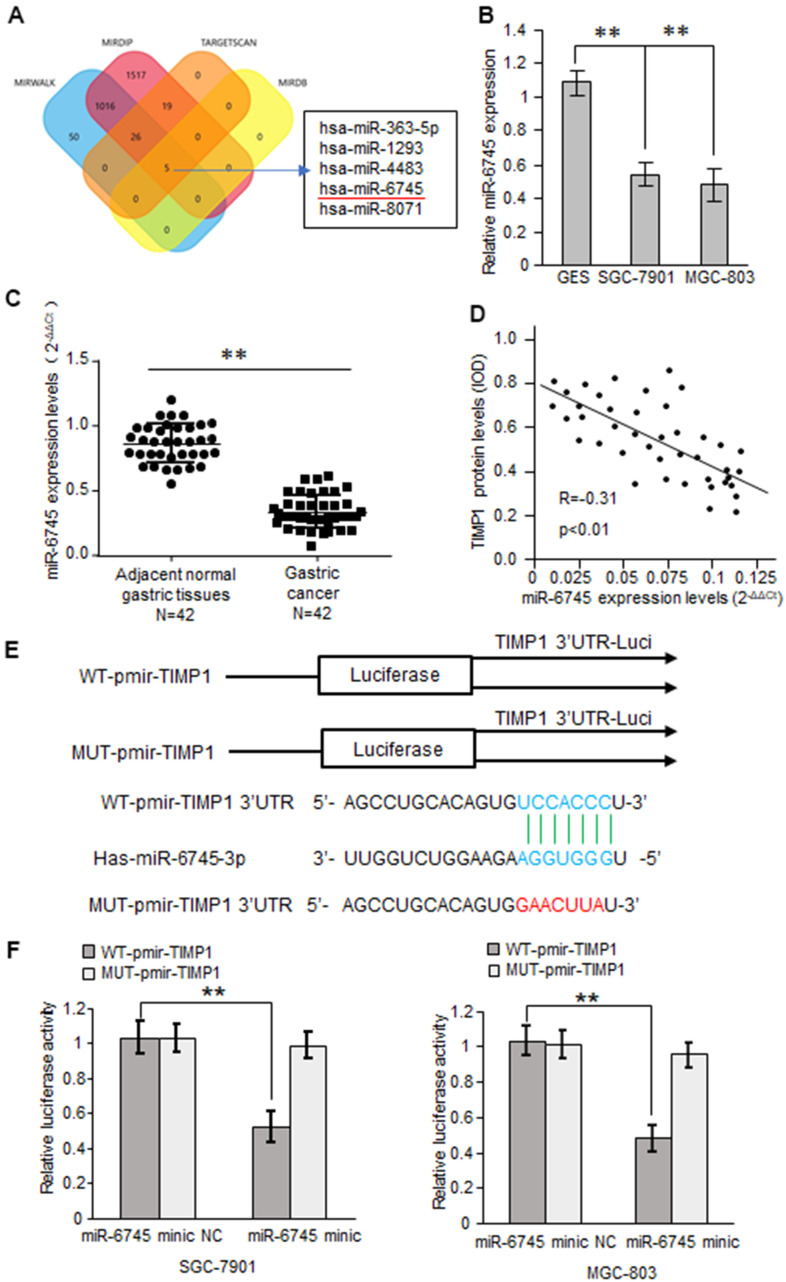
**TIMP1 is a direct target of miR-6745.** (**A**) The four-way Venn diagram reveals the numbers of overlapping miRNAs obtained using four publicly available bioinformatics algorithms and the microarray-based TIMP1 signature. (**B**) Real-time RT-PCR was used to detect the relative expression of miR-6745 in normal gastric cells and gastric cancer cells. (**C**) Analysis of miR-6745 expression in 42 gastric carcinoma tissues and 42 adjacent normal gastric tissues. (**D**) Correlation between miR-6745 levels and TIMP1 levels in 42 gastric carcinoma tissues. (**E**) Nucleotide predicted miR-6745-binding site in the TIMP1 mRNA 3′-UTR. (**F**) Luciferase activities were measured in SGC-7901 and MGC-803 cells transfected with reporter plasmids containing WT-pmir-TIMP1 or MUT-pmir-TIMP1 together with miR-6745 mimics or miR-6745 mimic NC. Data represent the means ± SEM. **P < 0.01.

Based on bioinformatics analysis, we predicted 5 potential binding miRNAs in TIMP1 3'-UTR (Figure 3A). Real-time RT-PCR results showed that the expression of miRNA-6745 was reduced in GC cells (Figure 3B). Through the detection of 42 cases of GC tissues and 42 adjacent normal gastric tissues, we found that miR-6745 was significantly reduced in GC tissues (Figure 3C). In addition, we found that TIMP1 protein levels were negatively correlated with miR-6745 levels in GC tissues (Figure 3D)*.* To explore whether miR-6745 directly binds to the TIMP1 3'-UTR, dual-luciferase reporter assays were performed (Figure 3E). The results showed that miR-6745 repressed luciferase activity of Wt-TIMP1 3′UTR, but not Mut-TIMP1 3′UTR (Figure 3F). These findings indicate that miR-6745 directly binds to the TIMP1 3'-UTR.

### miR-6745 inhibits GC proliferation, migration and invasion

**Figure 4:**
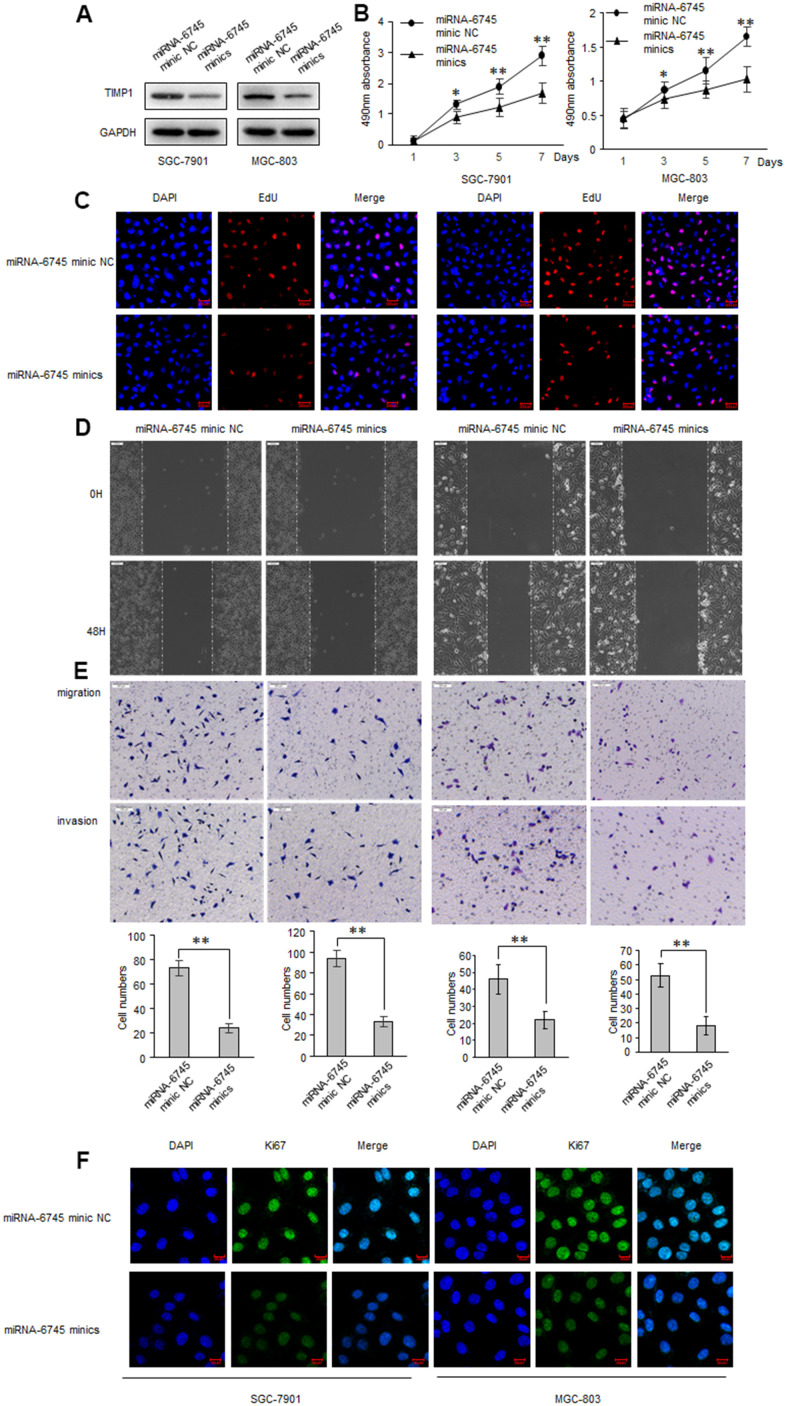
**miR-6745 inhibits migratory and invasive ability of GC cells.** SGC-7901 and MGC-803 cells were transduced with miRNA-6745 mimic NC or miRNA-6745 mimics. (**A**) Levels of TIMP1 were detected by western blot. (**B**) MTS assay indicted that miR-6745 mimics inhibited ability of proliferation. (**C**) Effect of miR-6745 on cell proliferative abilities was examined by EdU incorporation assay. (**D**) Cell would healing ability was impaired in miRNA-6745 mimics cells. (**E**) Chamber invasion ability was damaged in miRNA-6745 mimics cells. (**F**) The expression levels of Ki67 were detected by immunofluorescence. Data represent the means ± SEM. **P < 0.01.

TIMP1 promotes the GC process, and TIMP1 is the direct target of miR-6745, we then investigated whether miR-6745 have a similar effect on GC tumorigenesis? GC cells were transduced with miR-6745 mimics or miRNA-6745 mimic NC. Firstly, EdU incorporation assay and MTS assay were performed to assess GC cells growth and found that miR-6745 obviously decreased cell proliferation (Figure 4A–4C). Wound-healing and transwell assays were performed in GC cells, as shown in Figure 4D, 4E, the invasive and migratory ability were significantly reduced after transfected with miR-6745 mimics. Immunofluorescence staining also showed that miR-6745 also reduce the expression of KI67 (Figure 4F).

As expected, the cell proliferation invasive and migratory ability, and the expression of KI67 were significantly elevated when transfected with miR-6745 inhibitors (Supplementary Figure 2).

**Figure 5:**
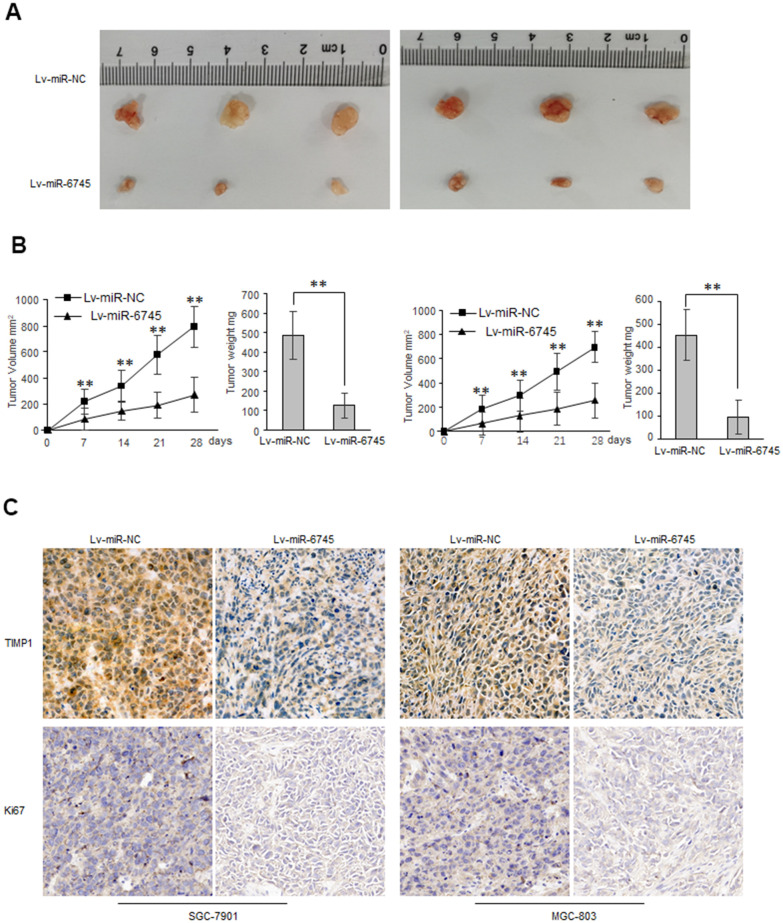
**miR-6745 suppresses gastric tumor growth *in vivo*.** Subcutaneous xenografts of GC cells infected with miR-6745 overexpressing lentivirus (Lv-miR-6745) or control lentivirus (Lv-miR-NC). (**A**) Images of the tumors at autopsy from nude mice are presented. (**B**) Tumor volumes and average weight of xenografted tumors were measured. (**C**) Immunohistochemical (IHC) staining of TIMP1 and Ki67 in xenografted tumors from Lv-miR-6745 cells or control cells. Data represent the means ± SEM. **P < 0.01.

To explore the biological functions of miR-6745 on GC cell proliferation and metastasis *in vivo*, we constructed two GC cell lines (Lv-miR-6745-SGC-7901 and Lv-miR-6745- MGC-803) that stably overexpresses miR-6745. Then we injected Lv-miR-6745 or Lv-miR-NC cells subcutaneously into nude mice. All mice were euthanized and stripped of tumor at 28 days after the experiment, the average tumor volumes and weights in Lv-miR-6745 group were smaller than Lv-miR-NC group (Figure 5A, 5B). Immunochemistry showed that that TIMP1 and Ki67 were significantly decreased in the Lv-miR-6745 group (Figure 5C). These results indicate that miR-6745 can inhibit tumorigenesis *in vivo*.

### miR-6745-TIMP1 axis regulates Wnt/β-catenin signaling in GC cells

**Figure 6:**
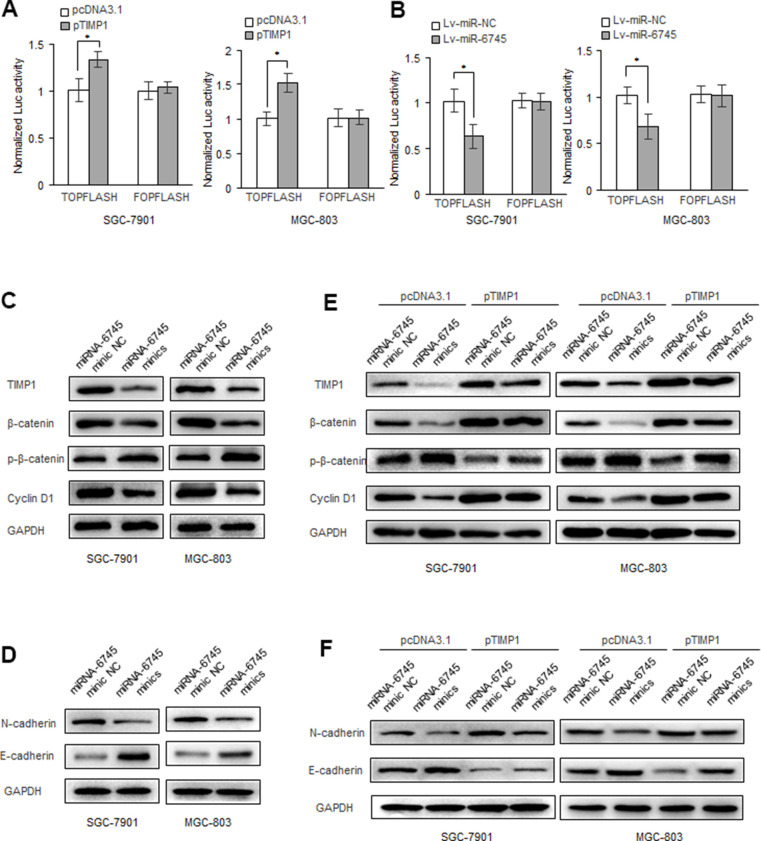
**miR-6745-TIMP1 axis regulates Wnt/β-catenin signaling in GC cells.** (**A**, **B**) β-catenin reporter assay in SGC-7901 and MGC-803 cells with TIMP1 overexpression (**A**) or miR-340 overexpression (**B**). (**C**, **D**) Effects of miR-6745 on protein levels of total β-catenin, phosphorylated β-catenin (Ser33/37/Thr41), cyclin D1, E-cadherin and N-cadherin. (**E**, **F**) TIMP1 partially restored the levels of total β-catenin, phosphorylated β-catenin (Ser33/37/Thr41), cyclin D1, E-cadherin and N-cadherin. Data represent the means ± SEM. **P < 0.01.

Overwhelming evidences had demonstrated that Wnt/β-catenin signaling play key roles in cancer proliferation, metastasis, and survival [18–20]. We found that TIMP1 can enhance the activity of the β-catenin reporter gene in GC cells (Figure 6A), while miR-6745 has the opposite effect (Figure 6B). And we found that miR-6745 overexpression or TIMP1 knockdown decreased the expression of total β-catenin in GC cells, and increased the level of phosphorylated β-catenin. In addition, we found that the overexpression of miR-6745 inhibited the levels of N-cadherin and cyclin D1 and increased the expression of E-cadherin (Figure 6C, 6D). And we found that TIMP1 can partially restore the expression of phosphorylated β-catenin, β-catenin, N-cadherin, E-cadherin and cyclin D1 regulated by miR-6745 (Figure 6E, 6F).

## DISCUSSION

In our study, we found that miR-6745 inhibits the proliferation, migration and invasion of GC cells. We demonstrated that miR-6745 inhibits the Wnt/β-catenin signaling pathway by reducing the expression of TIMP1, thereby reducing cell proliferation, migration and invasion *in vitro* and *in vivo.* Moreover, the analysis of GC tissues showed that the expression of miR-6745 in GC tissues was low, and it was negatively correlated with TIMP1.

TIMP1, which is the first-discovered natural collagenase inhibitor, has been demonstrated to be related to the occurrence of a variety of cancers [1, 21, 22]. TIMP-1 has both MMPs-dependent anti-proteolytic activity and MMP-independent cell growth activity [23]. Our results show that TIMP1 promotes the occurrence and development of GC, which is consistent with previous reports about TIMP1 as an oncogene in other cancers, including breast cancer [24], colon cancer [10], glioblastoma [25], and non-small cell lung cancer [26].

Wnt pathway is usually divided into classic Wnt signaling pathway (depending on β-catenin protein) and non-canonical Wnt signaling pathway (not dependent on β-catenin protein), which is highly conserved signaling pathways in cells [27, 28]. After the classical Wnt pathway is activated, cytoplasmic β-catenin escapes the degradation of GSK3β, the steady-state concentration increases, and β-catenin enters the nucleus. It can bind to transcription factors, especially TCF/LEF, to regulate the transcription of target genes [29]. This pathway mainly regulates cell differentiation and proliferation [30, 31]. Abnormally activated Wnt signaling pathway promotes the formation of pancreatic cancer and affects tumor metastasis [32, 33]. Our results show that in GC, overexpression of TIMP1 can activate the Wnt pathway. Abnormal expression of β-catenin plays an important role in the formation and development of GC [34, 35]. In addition, TIMP1 can also enhance the expression of CyclinD1 [36].

**Figure 7:**
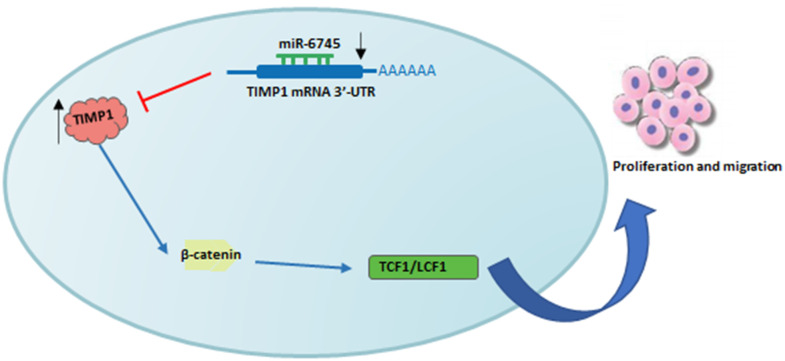
**Schematic diagram of roles of miR-6745-TIMP1 axis on Wnt/β-catenin signaling and its function in gastric tumorigenesis.** Under normal non-transformed conditions, miR-6745 binds to the 3-UTR in the TIMP1 mRNA and down-regulates TIMP1 protein levels. In GC, there is reduced levels of miR-6745 leading to elevated TIMP1 expression and activation of the Wnt/β-catenin signaling pathway.

In summary, we found that the downregulation of miR-6745 in GC tissues was negatively correlated with the high expression of TIMP1. As summarized in Figure 7, miR-6745 can inhibit the occurrence and development of GC by directly targeting TIMP1 to regulate downstream Wnt/β-catenin signaling. Therefore, we provide a new strategy for the prevention, diagnosis and treatment of GC.

## MATERIALS AND METHODS

### Tissue samples and cell lines

GC tissue samples and normal gastric tissue samples were collected from the Cancer Hospital of Hubei (Wuhan, P. R. China). This study obtained the informed consent of all patients and the approval of the Ethics Committee of the Cancer Hospital of Hube. MGC-803 and SGC-7901 cell lines were obtained from the Cell Bank of the Chinese Academy of Sciences (Shanghai, China). GC cells were grown in Roswell Park Memorial Institute (RPMI) 1640 medium (DMEM, BI, Israel) supplemented with 10% FBS and cultured at 37° C in a 5% CO_2_ humidified incubator.

### Transfection

GC cells were transfected with siRNAs, miRNAs, or plasmids using Lipofectamine 2000 (Invitrogen). miRNA mimics and miR-6745 inhibitor were obtained from RiboBio (Guangzhou, China). siTIMP1 was purchased from GenePharma (Shanghai, China). siRNA-TIMP-1 Target: ATCAACCAGACCACCTTATA; siRNA-Control Target: AATTCTCCGAACGTGTCACGT. The human TIMP1 overexpression plasmid TIMP1-bio-His was purchased from Addgene (Cambridge, MA).

### Real-time RT-PCR

RNA was extracted using TRIzol reagent (TaKaRa, Japan). 2 ug RNA was reverse-transcribed into cDNA with MLV-reverse transcriptase (Invitrogen), and Hieff qRT-PCR SYBR Green Master Mix was used for qRT-PCR (Tiangen, China). The primer sequences were as follows, TIMP1: 5'-CGCAGCGAGGAGGTTTCTCAT-3' and 5'- GGCAGTGATGTGCAAATTTCC-3'; GAPDH: 5'- ATGACATCAAGAAGGTGGTG -3' and 5'- CATACCAGGAAATGAGCTTG -3'.

### Western blotting

GC cells were lysed by RIPA buffer for 20min at 0° C. The proteins were processed by SDS-PAGE and transferred to PVDF membrane (BioRad). The primary antibodies used in Western blotting were as follows: anti-TIMP1 (ab211926; Abcam), anti-β-catenin (#8480; Cell Signaling Technology), anti-Ki67 (#9449; Cell Signaling Technology), anti-GAPDH (2188, Cell Signaling Technology), anti-p53 (10442-1-AP, Proteintech) and anti-phosphorylated β-catenin (Ser33/37/Thr41) (#9561, Cell Signaling Technology).

### Cell proliferation assay

Cell proliferation was detected using MTS Assay Kit (Sigma).

### Wound-healing assay

GC cells were seeded into 6-well plated and scratched with a pipette tip after an overnight incubation. Then, wash off the detached cells with PBS and continue incubating in serum-free medium.

### Transwell migration and invasion assays

Chamber invasion ability were performed with Corning chambers (Corning, USA). 1×10^5^ cells cultured with 200 μl serum-free media were seeded onto Transwell chambers. After incubating for 24 hours, cells were fixed with 4% polymethanol for 20 minutes, then stained with 0.1% crystal violet for 15 minutes, and pictures were taken under an inverted microscope for statistics.

### Immunofluorescence

GC cells were seeded into 12-well plated for 24 hours, and then transfected with siRNAs, miRNAs, or plasmids. After fixation with 4% paraformaldehyde, the cells were incubated with primary antibody at 4° C overnight. After washing 3 times with PBS the next day, the cells were incubated with fluorescently labeled secondary antibodies. A Zeiss confocal microscope was used for photo analysis.

### Animal studies

GC cells were transduced with a lentivirus expressing miR-6745 or a negative control (Genechem, Shanghai, China), and 500 ng/mL puromycin was used for selection. After 6 days, the cells were verified by real-time RT-PCR.

6-week-old male BALB/c nude mice were obtained from Beijing Vital River (Charles River Laboratories). And 5 × 10^7^ Lv-miR-6745 or Lv-miR-NC cells in 150 μL PBS were subcutaneously injected into the mice. The mice were euthanized after 28 days. All animal experiments were approved by Institutional Ethics Committee of Wuhan University of Science and Technology (P. R. China).

### Statistical analysis

All data are presented as means ± SEM. The experimental data were analyzed by SPSS v13.0. The statistical description of the experimental data in each group was represented by x±S. T-test and one way ANOVA with Tukey correction was used to analyze the presence of a difference among different groups. * P < 0.05. ** P < 0.01.

## Supplementary Material

Supplementary Figures
