# Supplementary material for: miR-6745-TIMP1 axis inhibits cell growth and metastasis in gastric cancer
Source: Aging (Albany NY). 2021 Nov 14;13(21):24402–16. doi: 10.18632/aging.203688 (PMC8610132; doi:10.18632/aging.203688)
Supplement: Supplementary Figures [file aging-13-203688-s001.pdf]

SUPPLEMENTARY FIGURES

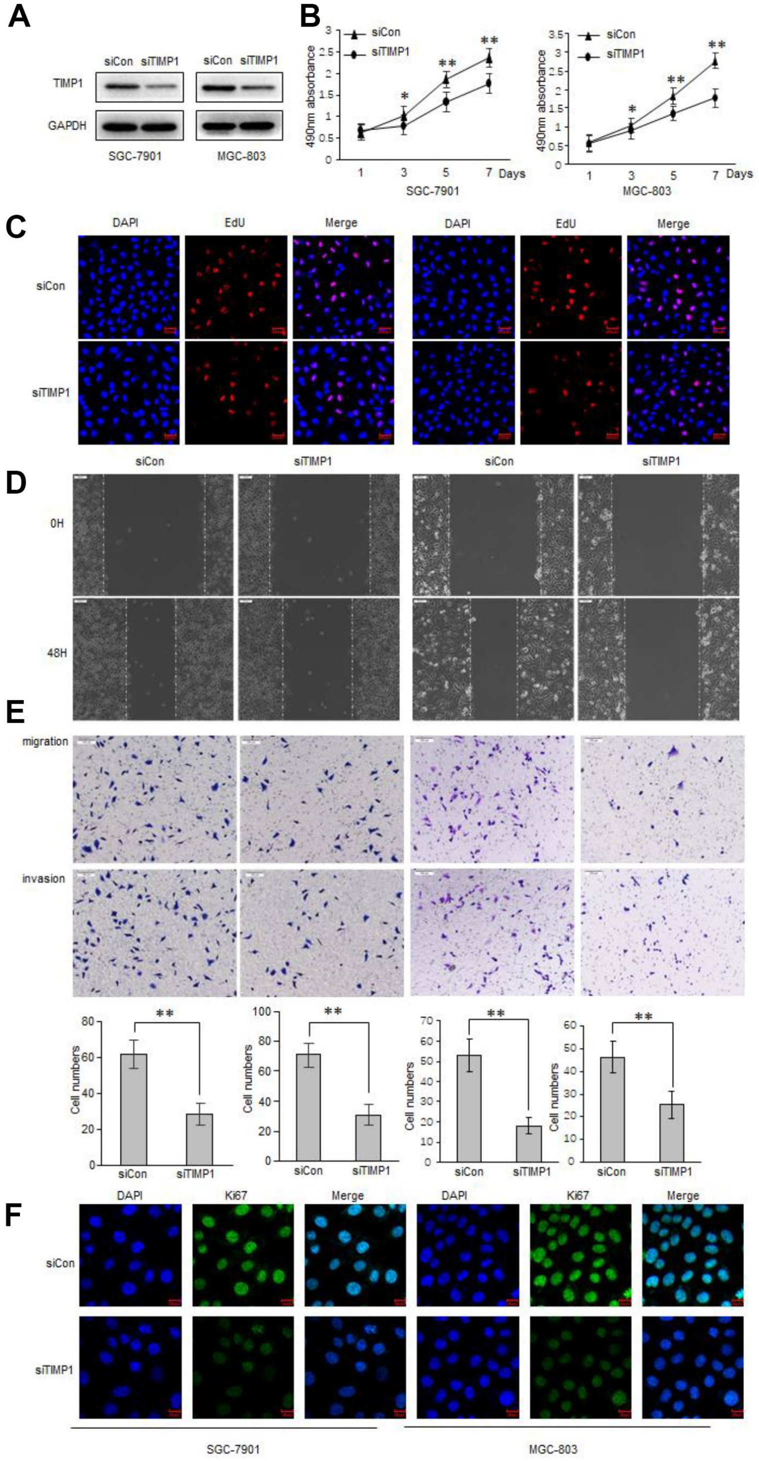

Supplementary Figure 1. Silencing TIMP1 reduces proliferation, migration, and invasion of GC cells. SGC-7901 and MGC-803 cells were transfected with siTIMP1 or siCon. (**A**) Levels of TIMP1 were detected by western blot. (**B**) Cell proliferation was determined by MTS assay. (**C**) Effect of miR-6745 inhibitor on cell proliferative abilities was examined by EdU incorporation assay. (**D**, **E**) Cell metastasis was determined by Scratch wound assays (**D**) or Transwell migration and Matrigel invasion assays (**E**). (**F**) The expression levels of the cell proliferation marker Ki67 were detected by immunofluorescence. Data represent the means ± SEM. **P < 0.01.

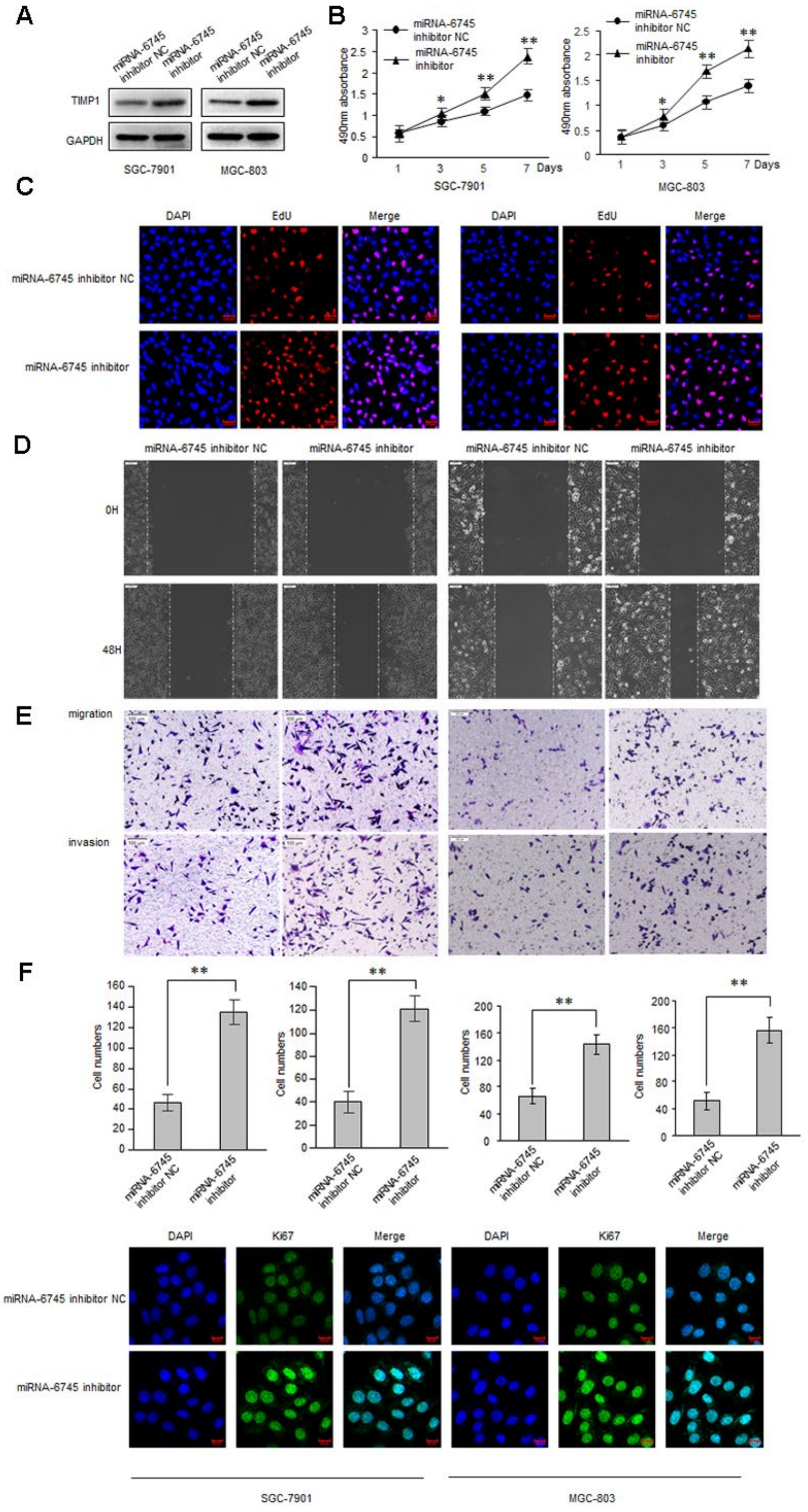

Supplementary Figure 2. miR-6745 inhibitor promotes migratory and invasive ability of GC cells. SGC-7901 and MGC-803 cells were transduced with miRNA-6745 inhibitor NC or miRNA-6745 inhibitor. (**A**) Levels of TIMP1 were detected by western blot. (**B**) MTS assay indicted that miRNA-6745 inhibitor promoted ability of proliferation. (**C**) Effect of miR-6745 inhibitor on cell proliferative abilities was examined by EdU incorporation assay. (**D**) Cell would healing ability was improved in miRNA-6745 inhibitor cells. (**E**) Chamber invasion ability was elevated in miRNA-6745 mimics cells. (**F**) The expression level of Ki67 was enhanced by immunofluorescence. Data represent the means ± SEM. **P < 0.01.
